# Draft Genome Sequence of Dyella sp. Strain C9, Isolated from a Malaysian Tropical Peat Swamp Forest

**DOI:** 10.1128/MRA.01083-18

**Published:** 2018-09-27

**Authors:** Chin Chin Too, Kuan Shion Ong, Sui Mae Lee, Catherine M. Yule, Alexander Keller

**Affiliations:** a School of Science, Monash University Malaysia, Bandar Sunway, Subang Jaya, Malaysia; b Tropical Medicine & Biology Multidisciplinary Platform, Monash University Malaysia, Bandar Sunway, Subang Jaya, Malaysia; c Department of Bioinformatics, University of Würzburg, Würzburg, Germany; d Center for Computational and Theoretical Biology, University of Würzburg, Würzburg, Germany; Georgia Institute of Technology

## Abstract

The bacterium Dyella sp. strain C9 was isolated from North Selangor Peat Swamp Forest, Malaysia, and studied using whole-genome sequencing. The putative genes involved in biogeochemical processes were annotated, and the genome sequence is publicly available in the NCBI database.

## Abstract (precis)

The bacterium Dyella sp. strain C9 was isolated from North Selangor Peat Swamp Forest, Malaysia, and studied using whole-genome sequencing.

## ANNOUNCEMENT

Tropical peat swamp forests (TPSF) are dense forests that are constantly waterlogged, acidic (pH 3 to 4), anoxic, and nutrient poor (1). In Southeast Asia, TPSF are found extensively in Indonesia and Malaysia. They are recognized as one of the largest terrestrial carbon sinks and are important in regulating global climate. Dyella sp. strain C9 was isolated from surface peat obtained from North Selangor Peat Swamp Forest, Malaysia, and its genome was sequenced to investigate putative functional genes involved in carbon and nitrogen cycles. Members of Dyella have been isolated from soil; for example, Dyella japonica was proposed to be capable of performing nitrate reduction and consuming glucose, fructose, and mannose (2), Dyella soli is potentially involved in nitrate reduction, and Dyella terrae can perform acetate assimilation (3). We previously sequenced the genome of Dyella sp. strain C11, which is potentially involved in the hydrolysis of cellulose, amylose, and hemicellulose and the production of a greenhouse gas, nitrous oxide (4).

Dyella sp. strain C9 was isolated in the same study that isolated Dyella sp. strain C11 (4). Briefly, the surface peat sample was enriched using primary enrichment medium (5), and bacterial isolation was performed on minimal medium using a common substrate in TPSF, methanol, as the carbon source (6). DNA extraction was conducted following the protocol of the QIAamp DNA minikit (Qiagen, Hilden, Germany), using a pure culture incubated at 30°C in tryptic soy broth for 36 h. PCR was performed targeted at 16S rRNA gene, followed by Sanger sequencing. The forward and reverse sequences were aligned in MEGA7 (7) and subject to a BLAST search in the EzBioCloud database (8), followed by a phylogenetic analysis in the maximum likelihood method using the most similar sequences; the organism was identified as a Dyella species. DNA library preparation was carried out following the protocol of the Nextera XT DNA library preparation kit (Illumina, San Diego, CA), and whole-genome sequencing was performed on the Illumina NextSeq 500 platform (2 × 150 bp) at the Biocenter of the University of Würzburg in Germany. SPAdes version 3.10.1 was used to correct DNA raw reads and perform *de novo* assembly with default settings (9). Contigs of <1,000 bp were removed using SeqFilter, followed by gene annotation using default settings in Prokka version 1.12 (10) and BlastKOALA (11). The Enzyme Commission (EC) numbers from the output of Prokka, and KEGG Orthologs (KO) assigned by BlastKOALA, were mapped to the metabolic pathways on the Kyoto Encyclopedia of Genes and Genomes (KEGG) database (12).

There were 623,158,818 raw reads, and 6,051,182 bp were assembled. There was a total of 43 contigs recovered, with a total length of 4,468,984 bp (*N*_50_, 195,450 bp), average sequencing depth of 139×, and 65.96% GC content. There were 4,994 coding DNA sequences (CDSs), 1 transfer-messenger RNA (tmRNA), 4 rRNAs, and 61 tRNAs found within the genome. The Dyella sp. strain C9 genome possessed the gene encoding the enzyme methane monooxygenase, which would enable it to perform methane oxidation; thus, it is potentially a methanotroph in nature. In addition, it was predicted to be involved in the degradation of phenols, ethanol, formate, pyruvate, starch, cellulose, hemicellulose, chitin, and sucrose. With regard to nitrogen metabolism, the gene nitrate reductase was observed, indicating its potential role in nitrate reduction to form nitrite.

### Data availability.

This whole-genome shotgun project has been deposited at DDBJ/ENA/GenBank under the accession number QIRF00000000. The version described in this paper is version QIRF01000000. Raw sequence reads were deposited in the Sequence Read Archive (SRA) under the accession number SRP157951.
